# Supplementary material for: Sustainable Ketalization of Glycerol with Ethyl Levulinate Catalyzed by the Iron(III)-Based Metal-Organic Framework MIL-88A
Source: Molecules. 2022 Oct 25;27(21):7229. doi: 10.3390/molecules27217229 (PMC9658270; doi:10.3390/molecules27217229)
Supplement: Supplementary file 1 [file molecules-27-07229-s001.zip › molecules-1852522-supplementary.pdf]

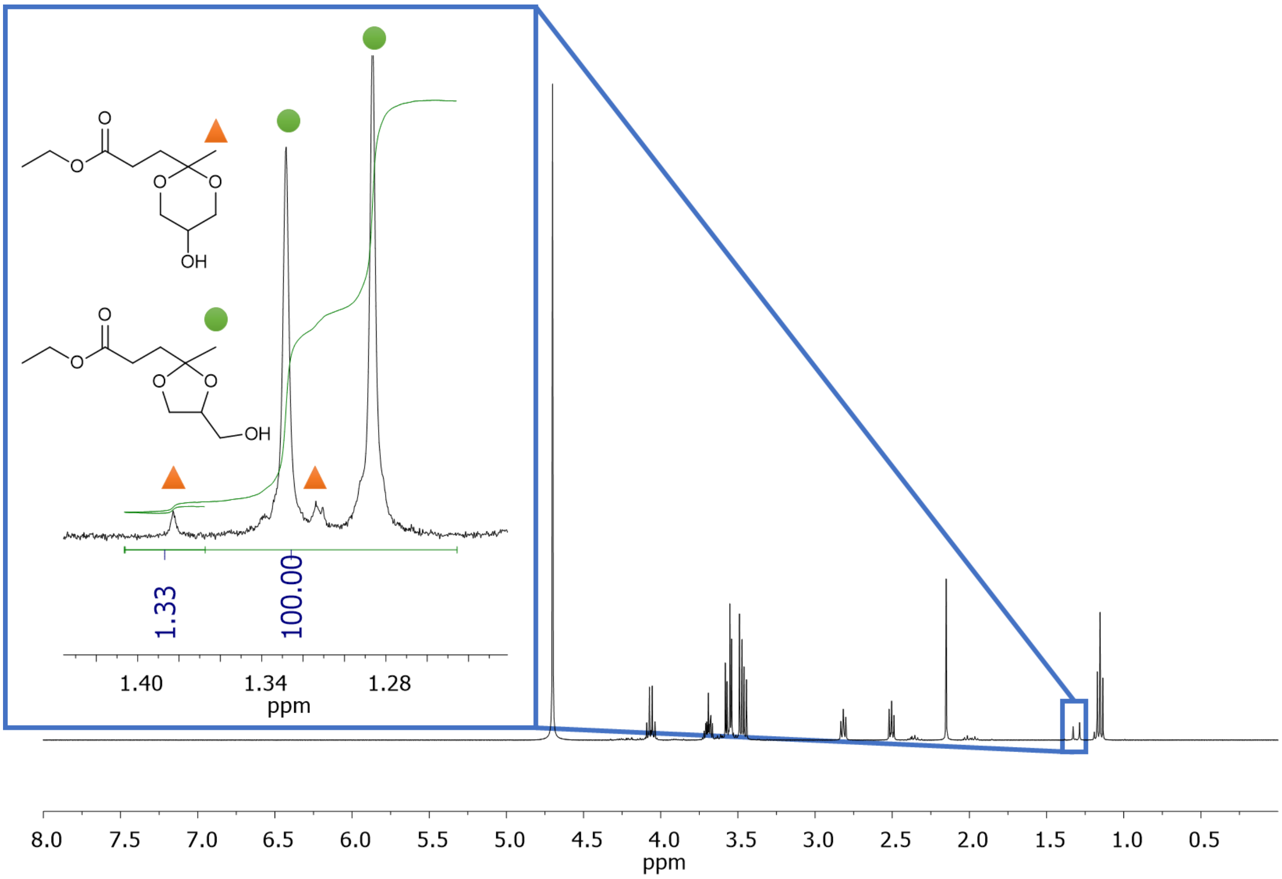

Figure S1. ^{1}H NMR spectra of crude reaction mixture in D_2O of Blank test at 22h

Table S1. Screening at a catalyst loading of 0.1% moles respect to glycerol.

Catalyst reuses	Yield at 22h[a]
Blank	64%
Run 0	78%
Run 1	77%
Run 2	79%

a) ^{1}H NMR Yield; errors within ±3%. Operative conditions: T=120°C, ethyl levulinate:glycerol= 1:1 mol/mol.

Table S2. Catalyst performances in 5 recycles.

**Catalytic runs**	***Yield*[a], %**								***[Fe(III)],***
	**0.25 h**	**0.50 h**	**1.0 h**	**2.0 h**	**3.0 h**	**4.0 h**	**6.0 h**	**22h**	**ppm[b]**
**Blank**	1	9	22	27	29	31	35	64	--
**Fe(III) 2 ppm**	7	17	21	25	27	29	33	64	--
**Run 0**	25	30	39	49	55	63	71	84	2±1
**Run 1**	24	30	35	44	61	66	74	83	<1
**Run 2**	25	31	32	43	53	60	71	84	<1
**Run 3**	23	28	39	46	53	67	73	85	<1
**Run 4**	25	28	37	49	65	70	76	87	<1
**Run 5**	23	30	35	45	53	59	69	84	<1

a) ^{1}H NMR Yields estimated errors within ±3%; b) evaluated by UV-Vis titration. Operative conditions: T=120°C, ethyl levulinate:glycerol= 1:1 mol/mol, catalyst load= 1% moles respect to glycerol.

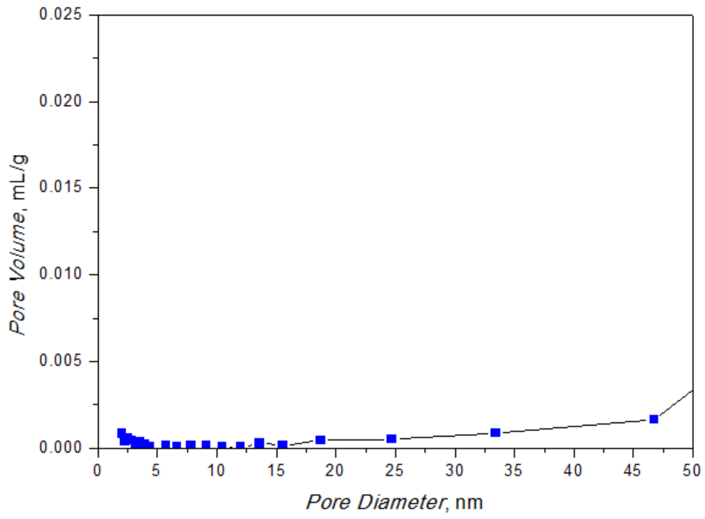

Figure S2. BJH analysis of MIL-88A.
